# Multi-Response Optimization of Drilling Parameters in Direct Hot-Pressed Al/B_4_C/SiC Hybrid Composites Using Taguchi-Based Entropy–CoCoSo Method

**DOI:** 10.3390/ma18184319

**Published:** 2025-09-15

**Authors:** Gokhan Basar, Funda Kahraman, Oguzhan Der

**Affiliations:** 1 Department of Industrial Engineering, Faculty of Engineering and Natural Sciences, Osmaniye Korkut Ata University, 80010 Osmaniye, Türkiye; 2 Department of Mechanical Engineering, Faculty of Engineering, Tarsus University, 33400 Mersin, Türkiye; fkahraman@tarsus.edu.tr; 3 Marine Engineering Department, Bandirma Onyedi Eylul University, 10200 Balikesir, Türkiye; oder@bandirma.edu.tr

**Keywords:** aluminium matrix hybrid composite, drillability, optimization, Entropy–CoCoSo method, Taguchi experimental design, hole quality characteristics

## Abstract

In this study, aluminium matrix hybrid composites reinforced with boron carbide (B_4_C) and silicon carbide (SiC) were fabricated using the direct hot-pressing technique under 35 MPa pressure at 600 °C for 5 min. Particle size distribution and scanning electron microscope analysis were conducted for the input powders. The microstructure, mechanical properties, and drillability of the fabricated composites were examined. As the SiC content increased, the density decreased, hardness improved, and transverse rupture strength declined. Drilling experiments were performed based on the Taguchi L_18_ orthogonal array. The control factors included cutting speed (25 and 50 m/min), feed rate (0.08, 0.16, and 0.24 mm/rev), point angle (100°, 118°, and 136°), and SiC content (0%, 5%, and 10%). Quality characteristics such as thrust force, torque, surface quality indicators, diameter deviation, and circularity deviation were evaluated. The Taguchi method was applied for single-response optimization, while the Entropy-weighted, Taguchi-based CoCoSo method was used for multi-response optimization. Analysis of Variance was conducted to assess factor significance, and regression analysis was used to model relationships between inputs and responses, yielding high R^2^ values. The optimal drilling performance was achieved at 50 m/min, 0.08 mm/rev, 136°, and 10% SiC, and the confirmation tests verified these results within the 95% confidence interval.

## 1. Introduction

Aluminium matrix hybrid composites (AMHCs) have emerged as high-performance engineering materials by combining the ductility and machinability of the aluminium (Al) matrix with the high hardness and strength of ceramic reinforcement phases [1]. Due to their advantageous features like light weight, high specific strength, thermal stability, and superior wear resistance, AMHCs have found widespread applications in aerospace [2], automotive, electronic [3], manufacturing [4], and marine industries [5]. These composites are particularly suitable for multifunctional material designs that demand simultaneous satisfaction of multiple performance requirements [1,6]. In this regard, lately, hybrid composites reinforced with more than one ceramic phase have drawn attention in scientific research-oriented bodies. Hybrid composites are currently produced with the synergy offered by boron carbide (B_4_C) and silicon carbide (SiC) powders.

Among the various production techniques for these composites, powder metallurgy stands out for its ability to provide homogeneous distribution of phases and microstructural control [7]. Specifically, the direct hot pressing (DHP) method enables the fabrication of dense, low-porosity composites with enhanced mechanical performance [8]. Operating under controlled temperature and pressure conditions, DHP offers a short processing time, improved particle bonding, and facilitates effective diffusion mechanisms during sintering [9]. Moreover, it ensures consistent and reproducible mechanical and microstructural properties in the produced composites [10].

After fabrication, machining operations—particularly drilling—are commonly required for assembling or integrating composite components [11]. However, the entity of hard and abrasive ceramic reinforcements in the matrix increases tool wear and reduces tool life [12]. Additionally, elevated cutting forces, heat generation, and vibrations during drilling can compromise the quality of the holes [13]. Therefore, assessing the machinability of AMHCs and determining the optimal drilling parameters are essential for ensuring dimensional accuracy, surface quality, and cost-effective manufacturing [14].

Drilling performance is affected by several parameters, including cutting speed (Vc), feed rate (f), point angle (PA), and reinforcement ratio (RR) [15]. These factors directly affect hole quality indicators such as thrust force (Fz), torque (Mz), surface roughness (Ra and Rz), diameter deviation (DD), and circularity deviation (CD) [16]. Hence, a quantitative estimation of the effect of every drilling parameter on such quality responses is pertinent to developing the whole process performance. So, with the help of a well-planned experimental and statistical setup, this work seeks to analyse the role of these parameters on all six-hole quality characteristics.

Although numerous studies in the literature have explored the drilling of AMHCs, many of them have predominantly focused on single-response evaluations, often limited to Fz or Ra and Rz. For instance, Okay et al. carried out the machinability of AMHCs reinforced with B_4_C and carbon nanofiber (CNT), analysing responses such as Fz, Mz, Ra, and delamination. However, their work, like many others, evaluated each response independently without integrating the outcomes into a holistic performance assessment [14]. Similarly, Senthil Babu et al. applied a drilling process to SiC and tungsten carbide reinforced Al7075 hybrid composites. The optimisation and modelling of drilling parameters were carried out. Their study identified optimal drilling conditions and modelled individual outputs such as Fz, Ra, and roundness error. Yet, the lack of a comprehensive multi-response evaluation limited the overall interpretability of trade-offs between conflicting parameters [17]. A more integrative perspective was attempted by Sapkota et al., who optimized the drilling of SiC, fly ash, and bagasse ash-reinforced AMHCs utilizing four different multi-objective decision-making techniques, including GRA and TOPSIS. Nevertheless, their work was still restricted to only two responses—material removal rate and Ra—thus missing additional critical indicators like delamination and dimensional deviations [18]. In another study, Kayaroganam et al. focused on Al/B_4_C/Mica composite and utilized modelling and optimization. Their research highlighted the significance of drilling parameters on Fz and Mz. While multi-objective in nature, the optimization was limited to just two quality responses, thus lacking a broader scope on overall drilling quality [19]. Franz et al. researched the influence of drilling parameters on surface quality, burr formation, and delamination in the drilling of fibre–metal laminates and hybrid stacks (such as CFRP/Al). However, their work was primarily a review and did not offer experimental validation or integrated modelling of multiple drilling outcomes [20]. Lastly, Yıldız and Sur studied the drilling behaviour of functionally graded AA7075/Al_2_O_3_ composites and used GRA to simultaneously evaluate Fz and Ra. While their approach was more comprehensive than earlier single-response methods, it still did not include critical outputs like delamination or dimensional accuracy [21]. Considering the above, it is clearly that while remarkable study has been dedicated to understanding drilling performance in AMHCs, a significant gap remains in studies that integrate multiple key quality indicators into a unified analytical framework. The novelty of the present study lies in its detailed and simultaneous evaluation of all principal drilling quality responses—Fz, Mz, surface quality indicators (Ra, Rz), and dimensional accuracy (DD, CD)—thereby providing a more complete understanding of the process behaviour and addressing a critical limitation in existing literature.

Although the Taguchi method is a straightforward, practical, and successful model for single-response optimisation, it is inadequate in multi-response scenarios where multiple conflicting objectives must be addressed. To overcome this limitation, a hybrid optimization approach—Taguchi-based Entropy–CoCoSo method—is adopted in this study. The Entropy method enables objective weighting of criteria based on data variability, while the CoCoSo method provides an integrated performance ranking. This hybrid strategy allows for effective and balanced multi-response optimization in drilling operations. Accordingly, this research consists of five major stages: (1) fabrication of Al/B_4_C/SiC hybrid composites using the DHP technique and characterization of their microstructure and mechanical properties, (2) experimental drilling tests and evaluation of six different hole quality indicators, (3) application of both single and multi-response optimization methods, (4) integration of Entropy-based weighting with the CoCoSo method, and (5) validation of the optimal drilling parameters through confirmation tests and confidence interval analysis. The scientific novelty of this study lies in the simultaneous optimization of six drilling quality indicators for Al/B_4_C/SiC AMHCs using an integrated Taguchi–Entropy–CoCoSo framework, and in mapping the composite performance index back to practical factor levels with experimental verification. This provides a validated and generalizable decision-making tool that extends beyond conventional single-response or unweighted approaches. Accordingly, this study provides a distinctive and thorough advancement to the domain of advanced composite machining and optimization.

## 2. Materials and Methods

### 2.1. Manufacturing of AMHC

In the manufacturing of composite materials, high-purity Al (>99%), B_4_C (>99.5%), and SiC (99.95%) powders were used. The particle sizes of the powders were measured using a Malvern Mastersizer 3000 device (Malvern Panalytical, Malvern, UK). The particle morphologies of the powders were analysed using a scanning electron microscope (SEM), specifically the Quanta 650 Field Emission model from FEI (Hillsboro, OR, USA). To produce composite materials with a pure Al matrix and a fixed 5 vol.% B_4_C content, 0, 5, and 10 vol.% SiC particles were added, and the powders were weighed accordingly. To the prepared powder mixtures, 1.5 wt.% of polyethylene glycol (PEG) 400 was added as a lubricant. The mixing process was conducted utilizing a Turbula mixer (Çelmak TB7, Elazığ, Türkiye) at a rotational speed of 40 rpm for 60 min. During mixing, one-third of the mixing container was filled with powder, another third with chrome-coated steel balls, and the remaining volume was left empty to provide uniform powder distribution. The mixed powders were placed into a graphite mold and consolidated utilizing a hot-pressing device equipped with programmable logic controller (PLC) control and direct resistance heating. For three-point bending specimens, a 40 × 10 × 10 mm graphite mold was used, whereas drilling specimens were consolidated in 40 × 40 × 10 mm graphite molds. All compacts were produced to near-net shape and lightly ground to achieve the final dimensions. The hot pressing was carried out under a pressure of 35 MPa, at 600 °C, for 5 min in an argon atmosphere. The experimental densities ($\rho_{e}$) of the samples were measured using Archimedes’ principle in accordance with the ASTM B962-17 standard [22]. The theoretical density ($\rho_{t}$) and relative density ($\rho_{b}$) of the composites were calculated using Equations (1) and (2), respectively [23].

(1) $$\rho_{t}=\rho_{m}{\times V}_{m}+\rho_{r}\times V_{r}$$

Here, $\rho_{m}$ and $V_{m}$ represent the density and volume fraction of the matrix powder, respectively, while $\rho_{r}$ and $V_{r}$ denote the density and volume fraction of the reinforcement particles.

(2) $$\rho_{b}=\frac{\rho_{e}}{\rho_{t}}\times 100$$

### 2.2. Microstructure and Mechanical Properties

**Figure 1:**
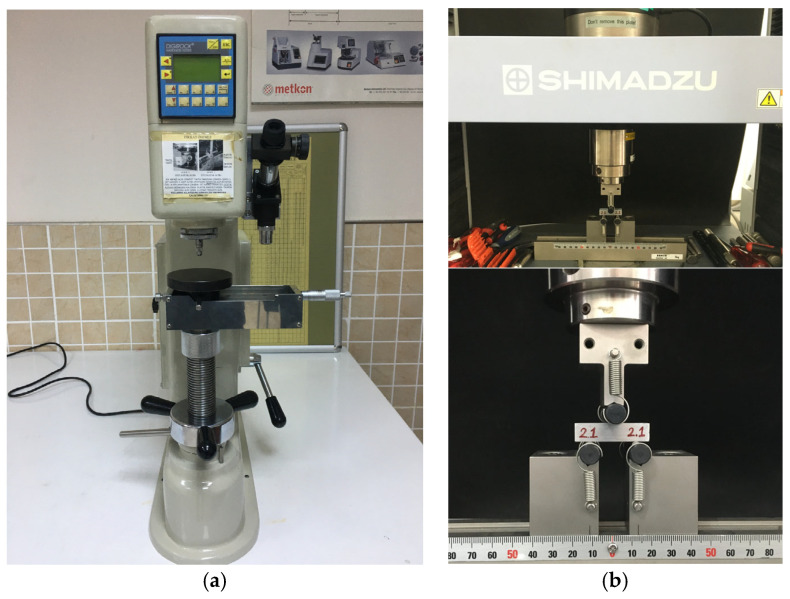
Devices used for mechanical property testing; (**a**) Brinell hardness tester, (**b**) Three-point bending test machine.

To characterize the microstructure of the AMHCs and to investigate the homogeneity of the reinforcement phase distribution, standard metallographic preparation was performed on the samples by applying grinding, polishing and etching processes in sequence. Subsequently, surface morphology and elemental composition analyses were performed using a SEM (ZEISS GeminiSEM 500, Oberkochen, Germany), and Energy Dispersive X-ray Spectroscopy (EDX) was conducted. Brinell hardness measurements were performed in accordance with the ASTM E10-17 standard using a 62.5 kgf load and a 2.5 mm ball indenter with the DIGI-ROCK-RBOV hardness tester (BMS Bulut Makina San. Tic. Ltd. Şti., Kocaeli, Türkiye) [24]. Three-point bending tests were performed in accordance with the ASTM B528-83a standard to establish the Transverse Rupture Strength (TRS) of the specimens produced with dimensions of 40 × 10 × 10 mm [25]. Bending tests were carried out using a Shimadzu Autograph AGS-X-100 kN testing device (Shimadzu Group, Kyoto, Japan). The tests were executed with a support span of 25 mm, and a loading rate of 0.5 mm/min. Bending tests were applied three times for each sample group. The average of the three test results was used to evaluate the TRS values. Equation (3) was used to calculate the TRS [26]. The devices used to evaluate the mechanical properties of the composites are shown in Figure 1.

(3) $$TRS=\frac{3Ul}{2t^{2}b}$$

Here, TRS denotes the transverse rupture strength (MPa); U is the applied load at fracture (N); l is the span length between supports (mm); t is thickness (mm); and b is the width of sample (mm).

### 2.3. Drilling Process

**Figure 2:**
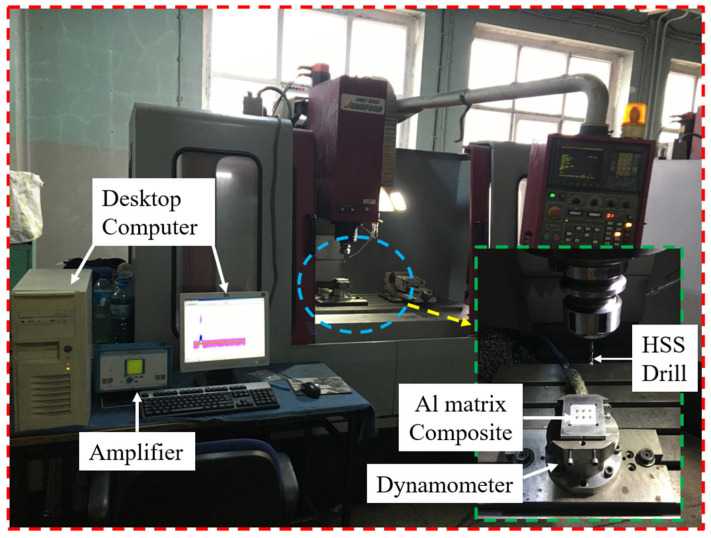
Experimental setup.

Drilling trials were conducted using a Johnford VMC-850 CNC vertical machining centre (JohnFord Roundtop Machinery Industries Co., Ltd., Taichung City, Taiwan), as shown in Figure 2. In the drilling tests, high speed steel (HSS) drills with a diameter of 5 mm and three different point angles (100°, 118°, and 136°) were used. The HSS drills employed in the experiments were supplied by Makine Takım Endüstrisi A.Ş. (Kocaeli, Türkiye). The 100°- and 136°-point angle HSS drills were obtained by regrinding standard DIN 338 HSS drills. The grinding of the drill point angles was performed with a tolerance of ±1–2°. A custom clamping fixture was designed and manufactured to mount the test specimens (40 × 40 × 10 mm) onto the dynamometer. This fixture was secured onto the dynamometer, which was itself mounted on the machine table. In this way, the drilling operations on the test specimens were performed in a more stable and reliable manner.

Drilling process of AMHCs, cutting speed, feed rate, point angle, and reinforcement ratio were selected as control factors, while Fz, Mz, Ra, Rz, DD, and CD were defined as quality characteristics. The control factors and levels were assigned based on literature studies and manufacturer recommendations, as presented in Table 1. The drilling tests of AMHCs were performed according to the mixed Taguchi L_18_ experimental design. Due to the ceramic particles contained in AMHCs, the cutting speed of HSS drills is limited. A review of studies in the literature shows that cutting speeds in the range of 25–67.5 m/min have been used [14,21]. Considering both the literature findings and the recommendations of the manufacturer, cutting speeds of 25 and 50 m/min were selected.

### 2.4. Measurement of Hole Quality Indicators

**Figure 3:**
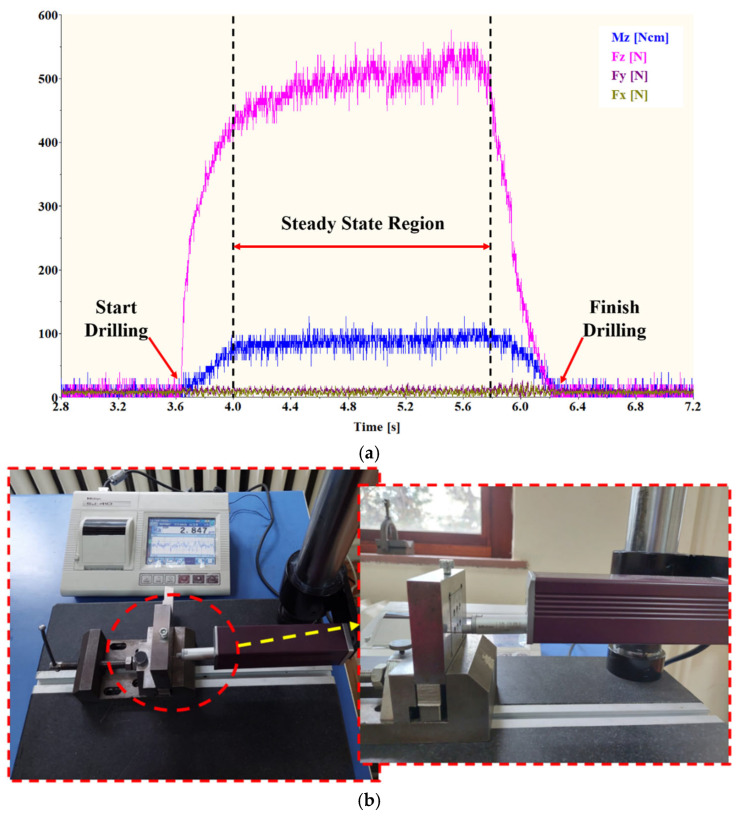

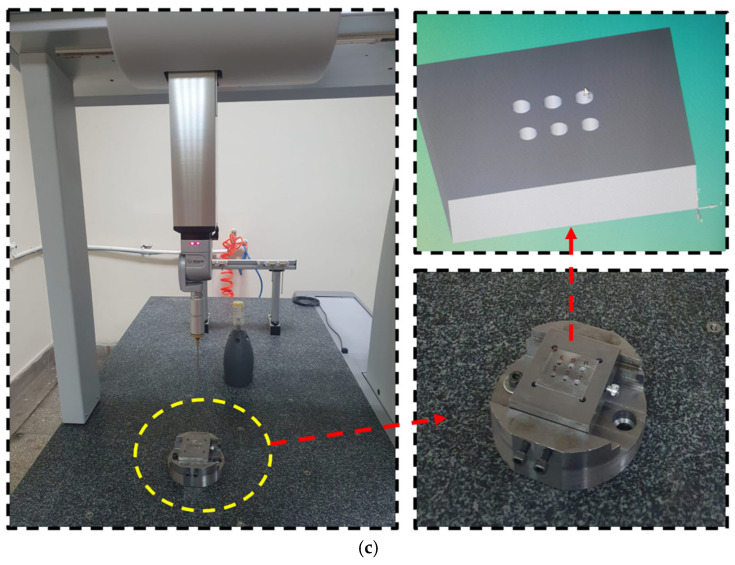
Measurement of hole quality indicators; (**a**) Fz and Mz, (**b**) Ra and Rz, (**c**) DD and CD.

During the drilling experiments, the Fz and Mz values were measured using a Kistler 9257A type dynamometer (Kistler Instrumente AG, Winterthur, Switzerland) mounted on a CNC vertical machining center. A Kistler 5070-A type multi-channel amplifier (Kistler Instrumente AG, Winterthur, Switzerland) was utilized to transfer the signals from the dynamometer to the data acquisition cards. The measurements obtained during the drilling tests were converted into numerical values using the DynoWare software (DynoWare type 2825D-02, Version 2.4.1.3, Kistler Group, Winterthur, Switzerland). The numerical values of the Fz and Mz were determined as the average values from the regions of the graph generated by the software where a consistent trend was observed. Drilling tests were performed three times, and the average values of the responses were calculated. The Mitutoyo Surftest SJ-410 Surface Roughness Tester (Mitutoyo, Kawasaki, Japan) was used for Ra and Rz measurements. These values were measured perpendicular to the holes, with a cutoff length of 0.8 mm and an evaluation length of 5, in accordance with ISO 4288:1996 [27]. For each specimen, a total of four measurements were taken by rotating the sample at 90° intervals. To determine the DD and CD, a Hexagon Global Performance coordinate measuring machine (CMM, Hexagon Metrology (Qingdao) Co., Ltd., Qingdao, China) was used. The DD and CD values of the holes were automatically measured at depths of −2 mm, −5 mm, and −7 mm along the *Z*-axis, taking the top surface of the material as the reference. The measurement images of the hole quality indicators are shown in Figure 3. A Dino-Lite AM4113T (AnMo Electronics Corporation, New Taipei City, Taiwan) digital optical microscope and Dino Capture 2.0 software were used to examine drill bits and obtain images of holes.

### 2.5. Taguchi Method

A loss function is used in the Taguchi method to determine the deviation between the observed and desired values [28]. This loss function is converted into a Signal-to-Noise (S/N) ratio and calculated according to the categories ‘Smaller-the-better’, ‘Larger-the-better’ and ‘Nominal-the-best’ using Equations (4)–(6), respectively [29,30]. In this study, since the objective values in the optimization of hole quality indicators were to be minimized, the criterion ‘Smaller-the-better’ was applied and Equation (4) was used accordingly.

**In the case of “Smaller-the-better”:**

(4) $$\frac{S}{N}=-10log\left(\frac{1}{n}\sum_{i=1}^{n}y_{i}^{2}\right)$$

**In the case of “Larger-the-better”:**

(5) $$\frac{S}{N}=-10log\left(\frac{1}{n}\sum_{i=1}^{n}\frac{1}{y_{i}^{2}}\right)$$

**In the case of “Nominal-the-best”:**

(6) $$\frac{S}{N}=10log\left(\frac{{\bar{y}}^{2}}{S^{2}}\right)\;\bar{y}=\frac{1}{n}\sum_{i=1}^{n}y_{i}\;S^{2}=\frac{1}{n-1}\sum_{i=1}^{n}{\left(y_{i}-\bar{y}\right)}^{2}$$

Here; $y_{i}$: denotes the ith value of the quality attribute, n: denotes the number of tests, $\bar{y}$: denotes the average of the values, and $S^{2}$ denotes the variance of the observed values [30].

The highest S/N ratio calculated for objective function indicates the best experimental result. The optimal experimental condition is the level of the control factors with the highest S/N ratio [31].

### 2.6. Entropy Method

In decision-making problems involving multiple performance criteria, the determination of criterion weights is of great importance [32]. In the literature, various subjective and objective methods have been proposed to determine the weights of performance criteria. Among these, the entropy method is classified as an objective weighting technique [33,34]. The application steps are presented below in sequence.

Step 1: Determination of the Decision Matrix:

In Multi-Criteria Decision-Making (MCDM) problems, a decision matrix X, consisting of m alternatives and n criteria, is shown in Equation (7). This matrix X is composed of the values that the alternatives receive with respect to each criterion.

(7) $$X=x_{ij}=\left[\begin{array}{ccc} x_{11} & \ldots & x_{1\ddot{n}}\\ \vdots & \ddots & \vdots \\ x_{\ddot{m}1} & \ldots & x_{\ddot{m}\ddot{n}} \end{array}\right]$$

i = 1, 2, 3, … $\ddot{m}$ represents the alternatives, and j= 1, 2, 3, … $\ddot{n}$ represents the evaluation criteria. $x_{ij}=$ j. denotes the value of the ith alternative with respect to the jth evaluation criterion.

Step 2: Obtaining the normalized decision matrix values:

To make the criteria with different units of measurement in the decision matrix comparable, a normalization process must be applied. The normalized values denoted as $r_{ij}$ are calculated using Equation (8).

(8) $$r_{ij}=\frac{x_{ij}}{\sum_{i=1}^{\ddot{m}}x_{ij}}$$

Step 3: Obtaining the entropy coefficient values:

The entropy coefficient values ($e_{j}$), for all criteria are calculated using Equations (9) and (10).

(9) $$e_{j}=-k\sum_{j=1}^{\ddot{n}}r_{ij}\ln r_{ij}$$

(10) $$k=\frac{1}{\ln\ddot{n}}$$

Step 4: Obtaining the degree of diversification values:

The degree of diversification ($d_{j}$), for all criteria is calculated using Equation (11), based on the entropy values obtained in the previous step.

(11) $$d_{j}=\left|1-e_{j}\right|$$

Step 5: Calculation of the entropy weights:

The entropy weight $w_{j}$ for all criteria is calculated using Equation (12). The entropy weight indicates the relative importance of each criterion. As the entropy coefficient decreases, the entropy-based weight increases. In other words, the corresponding criterion provides more information and plays a more significant role in the decision-making process compared to the other criteria.

(12) $$w_{j}=\frac{d_{j}}{\sum_{j=1}^{\ddot{n}}d_{j}}$$

### 2.7. Taguchi-Based Combined Compromise Solution (CoCoSo) Method

The CoCoSo method emerged because of integrating two MCDM methods: SAW (Simple Additive Weighting) and EWP (Exponentially Weighted Product). This approach was developed in 2019 by Yazdani, Zarate, Zavadskas, and Turskis [35]. In this approach, the benefit values of the decision alternatives are initially determined from multiple perspectives using various aggregation operators. These benefit values of the alternatives are then combined using a compromise solution and an aggregation function. The method offers populating a ranking of the decision alternatives with great stability, robustness, and reliability. Compared to other MCDM methods, the rankings from CoCoSo are less affected by the addition or removal of a new alternative in the decision matrix. Because of its ability to derive reliable results and being applicable in scientific studies, the method presents a different viewpoint for decision-makers in working with decision-making problems [36].

In the drilling experimental context, process parameters’ levels were less than optimum for multi-response optimization, as determined by the Integration of the CoCoSo method with the Taguchi method. On the other hand, this hybrid technique called the Taguchi-based CoCoSo method exploits the advantages of both approaches to improve the robustness and the reliability of the optimization technique. These steps for the implementation have been laid out extensively, with the first five steps coinciding with the usual CoCoSo method, while Step 6 incorporates the Taguchi method for the analysis of means and finds the optimal levels of process parameters. The union of these two methodologies thus gives a comprehensive and practical framework to tackle intricate multi-response decision-making problems faced in drilling applications.

Step 1: Construction of the decision matrix

First, the decision maker constructs a decision matrix consisting of the values assigned to the alternatives based on each criterion.

Step 2: Normalization of benefit and cost criteria

To make the criteria with different units of measurement in the decision matrix comparable, a normalization process must be applied. Equation (13) is used for the normalization of benefit-based criteria, while Equation (14) is used for the normalization of cost-based criteria. Here, $minx_{ij}$ represents the minimum value, and $maxx_{ij}$ represents the maximum value.

(13) $$r_{ij}=\frac{x_{ij}-minx_{ij}}{maxx_{ij}-minx_{ij}}\;\mathrm{for benefit-based criterion}$$

(14) $$r_{ij}=\frac{maxx_{ij}-x_{ij}}{maxx_{ij}-minx_{ij}}\;\;\mathrm{for cost-based criterion}$$

Step 3: Calculation of the $S_{i}$ and $P_{i}$ values

The $S_{i}$ values based on the grey relational approach and the $P_{i}$ values based on the multiplicative property of the WASPAS method are calculated using Equations (15) and (16), respectively. The $w_{j}$ in the equations indicate the weight of each criterion.

(15) $$S_{i}=\sum_{j=1}^{\ddot{n}}\left(w_{j}r_{ij}\right)$$

(16) $$P_{i}=\sum_{j=1}^{\ddot{n}}{\left(r_{ij}\right)}^{w_{j}}$$

Step 4: Calculation of the relative performance scores of the decision alternatives

The relative performance scores of the decision alternatives ($k_{ia},k_{ib},k_{ic}$) are calculated using Equations (17)–(19), respectively.

(17) $$k_{ia}=\frac{P_{i}+S_{i}}{\sum_{i=1}^{\ddot{m}}\left(P_{i}+S_{i}\right)}$$

(18) $$k_{ib}=\frac{S_{i}}{minS_{i}}+\frac{P_{i}}{minP_{i}}$$

(19) $$k_{ic}=\frac{\lambda \left(S_{i}\right)+\left(1-\lambda \right)\left(P_{i}\right)}{\left(\lambda maxS_{i}+\left(1-\lambda \right)maxP_{i}\right)}$$

The λ value in Equation (19) is generally selected as 0.5 in the literature. However, the choice of λ is left to the decision maker’s preference.

Step 5: Calculation of the performance indicator of the decision alternatives:

The performance indicator of the decision alternatives ($k_{i}$), is calculated using Equation (20) based on the relative performance scores given in Equations (17)–(19).

(20) $$k_{i}={\left(k_{ia}k_{ib}k_{ic}\right)}^{\frac{1}{3}}+\left(k_{ia}+k_{ib}+k_{ic}\right)$$

Step 6: Determination of the optimal experimental parameters:

The $k_{i}$ values obtained according to the Taguchi orthogonal array are converted into S/N ratios for evaluation. The conversion of $k_{i}$ values into S/N ratios is performed using the “larger-the-better” objective function. The $k_{i}$-S/N values are calculated using Equation (5). In the main effects plot for the S/N ratios of the performance indicator of the decision alternatives, the levels with the highest S/N values indicate the optimal experimental parameters.

## 3. Results and Discussion

### 3.1. Properties of Powders

**Figure 4:**
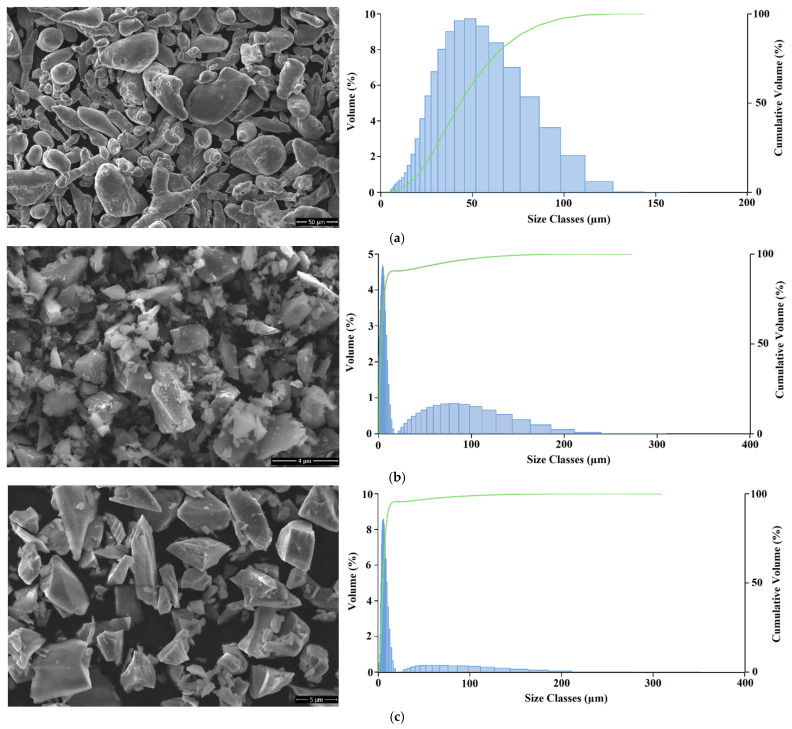
Particle size distributions and SEM images (×10,000 magnification) of the powders; (**a**) Al, (**b**) B_4_C, (**c**) SiC.

The SEM images and particle size distributions of the raw powders used in the production of AMHCs are presented in Figure 4. It is acquired that the Al powder particles exhibit round, rod-like, spherical, and irregular shapes, while the B_4_C powder particles have irregular and sharp-edged shapes, and the SiC powder particles are also irregular and sharp-edged. An examination of the particle sizes of the powders reveals that the Al powder is highly heterogeneous. The D10 value of 20.3 µm and the D90 value of 79.1 µm indicate a wide variation in particle sizes, while the D50 value is 43.3 µm. In contrast, the B_4_C powder exhibits an extremely fine and narrow distribution. With a D50 value of 2.84 µm, its D10 and D90 values are 0.523 µm and 12.6 µm, respectively. These results show that while most particles range from sub-micron size up to approximately 13 µm, the main population is concentrated within a much narrower interval. The SiC powder demonstrates intermediate characteristics among the three powders analyzed. Compared with B_4_C, it is coarser; however, it is much finer and more homogeneous than Al powder. The D50, D10, and D90 values of SiC are 5.08 µm, 2.11 µm, and 10.8 µm, respectively, indicating a relatively narrow and uniform size distribution. In summary, B_4_C has the finest and most homogeneous distribution, SiC shows medium fineness and homogeneity, while Al displays the coarsest and most heterogeneous distribution.

### 3.2. Density, Microstructure and Mechanical Properties

The density measurement results of the composites are presented in Table 2. According to these results, increasing the SiC reinforcement ratio significantly increases porosity in the composites. The main reason for these situations is that high SiC reinforcement levels, poor wettability between the hard ceramic particles and the Al matrix, create a diffusion barrier at the interface region [37]. The thermal conductivity of SiC (120 W/mK) [38] hinders heat transfer within the composite, thereby slowing down diffusion mechanisms during the sintering process [39]. Moreover, the high hardness of SiC particles (25.7 ± 1.2 GPa) [40] prevents plastic deformation during powder compaction.

**Figure 5:**
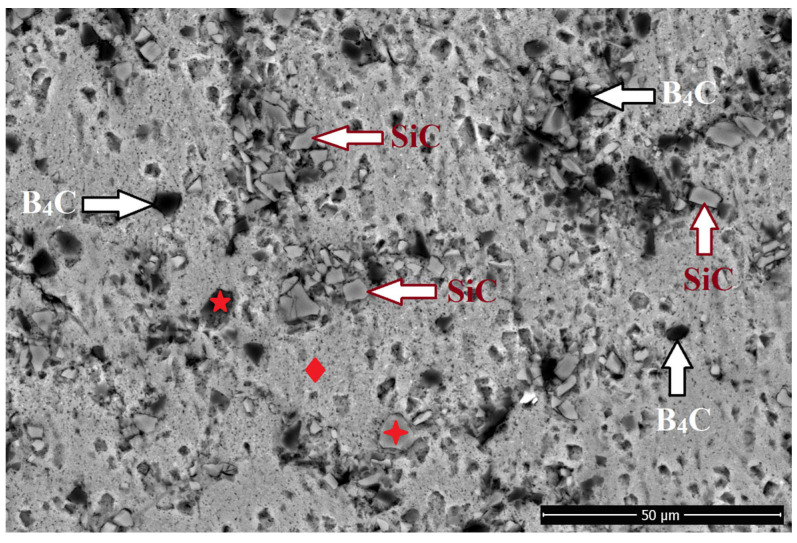

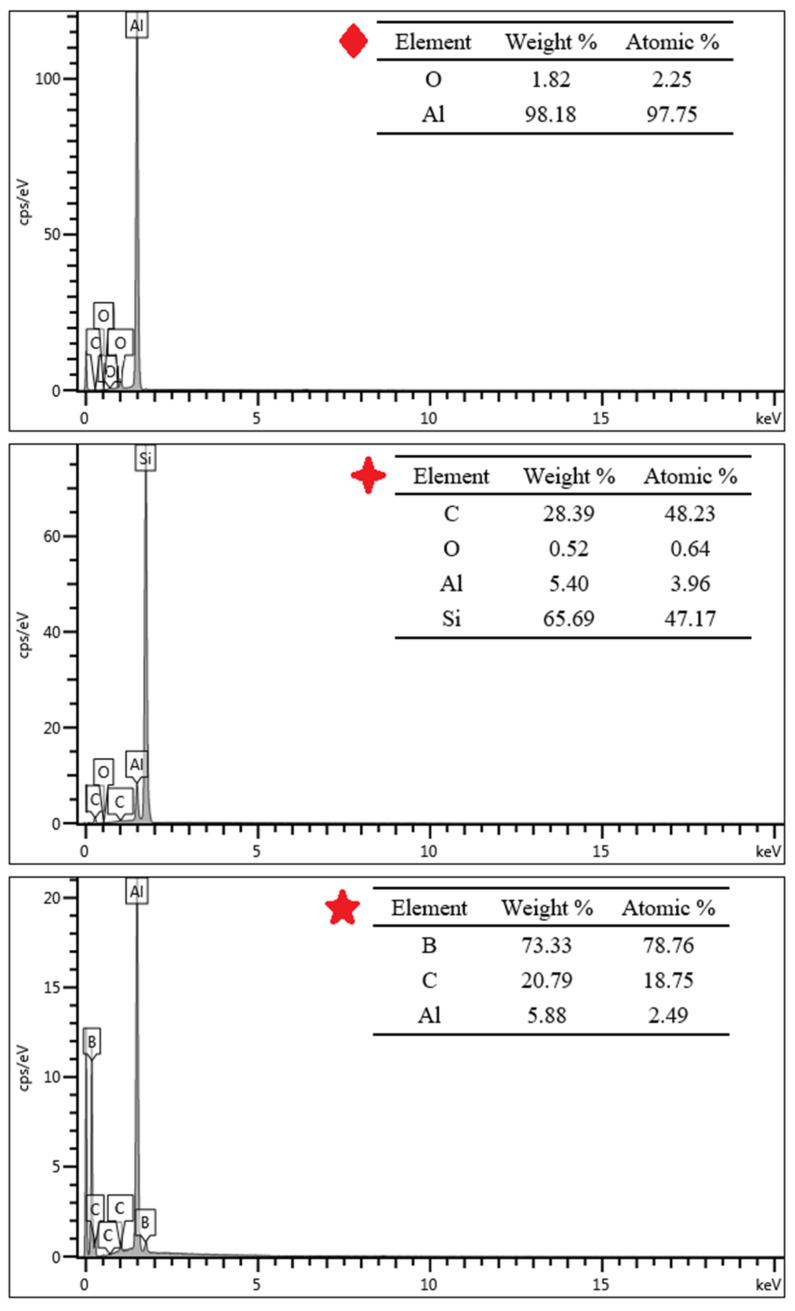
The SEM/EDX analysis in the microstructure of Al/5B_4_C/5SiC.

The EDX/SEM analysis results of the microstructure of Al/5B_4_C/5SiC are presented in Figure 5. Upon examination of the figure, it was determined that the bright areas in the SEM image correspond to SiC ceramic particles, while the dark areas represent B_4_C ceramic particles. Additionally, the SiC and B_4_C ceramic particles exhibited a partially homogeneous distribution within the matrix. However, in certain regions, agglomeration of ceramic particles was observed. Local clustering of hard ceramic particles within the aluminum matrix adversely affects the mechanical properties of the composite. The primary reason for this effect is the formation of stress concentrations, which hinder effective load transfer across the matrix and cause irregularities in load distribution [41]. These clusters also weaken the matrix–reinforcement interfacial bonds, while the associated stress fields accelerate crack propagation and ultimately lead to premature failure [42]. Nazik et al. [43] reported that increasing the volume fraction of B_4_C reinforcement promotes clustering, which weakens interfacial bonding and produces adverse effects that outweigh the potential performance improvements of the composite. Similarly, Suarsana and Soenoko [44] demonstrated that SiC clustering in the microstructure impairs load transfer, leading to localized damage and reduced ultimate tensile strength. In line with these findings, Eskandari et al. [45] showed that SiC clusters facilitate stress accumulation under load, thereby accelerating early crack initiation and propagation and resulting in decreased flexural strength.

**Figure 6:**
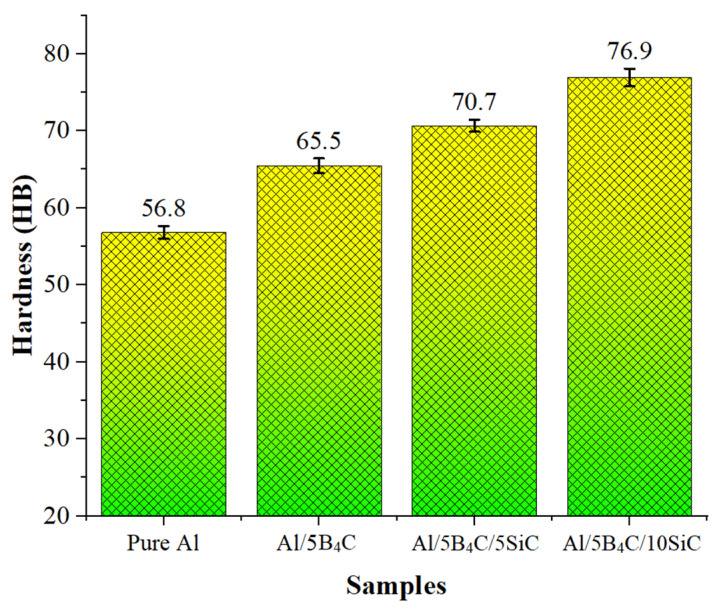
Hardness values.

The average Brinell hardness values of the samples are presented in Figure 6. The hardness values were determined as 56.8 HB for pure Al, 65.5 HB for Al/5B_4_C, 70.7 HB for Al/5B_4_C/5SiC, and 76.9 HB for Al/5B_4_C/10SiC, respectively. The hard B_4_C ceramic particle reinforcement in the Al/5B_4_C composite material increased the hardness of pure Al by 15.32%. In the Al/5B_4_C/5SiC AMHC material, the hard SiC ceramic particle reinforcement further increased the hardness by 7.94% compared to the Al/5B_4_C composite. It has been observed that hardness values increase with the increase in SiC particle RR, and the results are consistent with the findings reported in the literature [46,47,48]. The increase in hardness with the rising SiC reinforcement ratio is attributed to the residual stresses generated due to the difference in the coefficients of thermal expansion between the matrix and the reinforcing elements. These thermally induced residual stresses hinder the movement of dislocations, thereby contributing to the strengthening of the composite structure. Additionally, this effect, as demonstrated in several studies in existing literature, the uniform distribution of SiC particles within the Al matrix ensures effective load transfer and enhances hardness. Homogeneous dispersion of SiC particles not only improves stress distribution but also plays an important role in enhancing the overall mechanical properties of the composite material, particularly its hardness [49,50].

**Figure 7:**
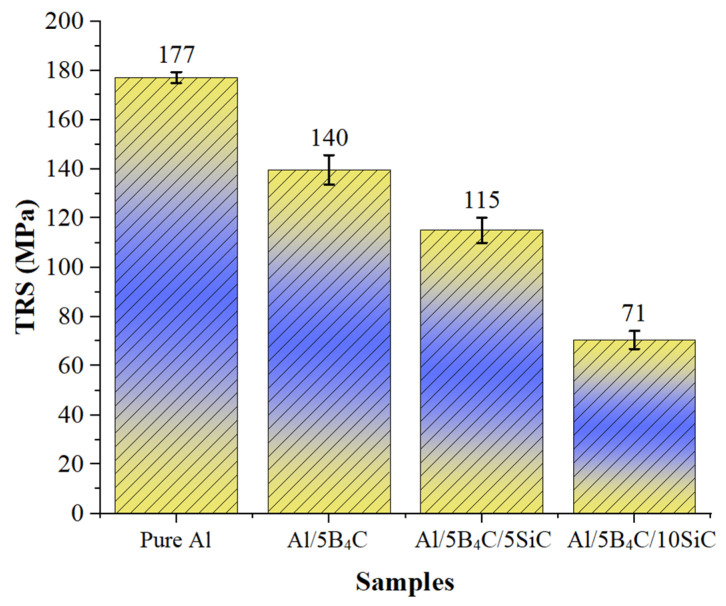
Average TRS values of the specimens.

The average TRS values of the AMHCs are shown in Figure 7. The calculated TRS values were determined to be 177 MPa for pure Al, 140 MPa for Al/5B_4_C, 115 MPa for Al/5B_4_C/5SiC, and 71 MPa for Al/5B_4_C/10SiC. The TRS value of pure Al decreased by 20.90% in the Al/5B_4_C composite material reinforced with B_4_C ceramic particles. In the Al/5B_4_C/5SiC AMHC material, produced with the addition of SiC as a second hard ceramic particle, the TRS value showed a further reduction of 17.86% compared to the Al/5B_4_C composite. It was obtained that the TRS values decreased with the increase in the SiC particle RR. The increase in the RR led to a decrease in relative density and an increase in porosity, which negatively affected the TRS values. This is because an increase in porosity results in a reduction in the cross-sectional area of the composite materials, leading them to fracture under lower loads. The results obtained are consistent with the literature [51,52]. Arık produced a composite material with a pure Al matrix and 10% SiC reinforcement. When comparing the mechanical properties of the SiC-reinforced composite with those of pure Al, it was noted that the hardness of the SiC-reinforced composite increased, while its TRS value decreased [53]. In the study by Okay and Islak, it was reported that AMHCs reinforced with B_4_C and CNFs, produced by the hot-pressing method, an increase in CNF reinforcement led to an increase in hardness values, while the TRS results decreased due to the rise in porosity [51]. Additionally, it was stated that the increase in flake reinforcement content created a notch effect and reduced the TRS values [54].

It is noteworthy that while the hardness values increase with higher SiC reinforcement ratios (Figure 6), the TRS exhibits an opposite decreasing trend (Figure 7). This behavior can be explained by the fact that SiC particles, due to their intrinsic hardness and thermal expansion mismatch with the Al matrix, promote strengthening through residual stresses and effective load transfer [37]. However, the higher reinforcement content also leads to increased porosity and weaker interfacial bonding, thereby reducing the effective load-bearing cross-sectional area [55]. These microstructural deficiencies facilitate early crack initiation and propagation, resulting in reduced bending strength despite the hardness improvement. Such a trade-off between hardness and transverse rupture strength is well-documented in ceramic-reinforced aluminum composites.

### 3.3. Drilling of AMHCs

Single- and multi-response optimization processes have been applied to improve hole quality while drilling AMHCs. Single-response optimization for each output was carried by means of the Taguchi technique. On the other hand, the multi-response optimization was realized by employing the Taguchi-based Entropy–CoCoSo method. This method determines the weights of the hole quality indicators according to the Entropy method. Furthermore, ANOVA analyses and regression analyses were conducted. The expert experiments were carried out finally, followed by calculating the confidence intervals.

#### 3.3.1. S/N Analyses

The drilling process of AMHCs was performed according to the Taguchi L_18_ experimental design. To determine hole quality, measurements of Fz, Mz, Ra, Rz, DD, and CD were conducted. Additionally, S/N ratios were computed for each response to identify the optimum drilling conditions. The measurement results of the hole quality indicators and the computed S/N ratios are presented in Table 3. To improve hole quality, S/N ratios were calculated for each response using Equation (4) based on the “smaller-is-better” objective function. When Table 3 is examined, the lowest values of Fz, Mz, DD, and CD and their corresponding S/N ratios were obtained under experimental condition No. 3 (Vc: 25 m/min, f: 0.08 mm/rev, PA: 136°, RR: 10%). The lowest Ra and Rz values and their corresponding S/N ratios were obtained under experimental condition No. 10 (Vc: 50 m/min, f: 0.08 mm/rev, PA: 100°, RR: 10%). The average response values and corresponding average S/N ratios measured in the experiments were determined as follows: the average Fz value was 491.111 N with an average S/N ratio of −52.905 dB, the average Mz value was 89.722 N·cm with an average S/N ratio of −38.368 dB, the average Ra value was 4.630 µm with an average S/N ratio of −13.095 dB, the average Rz value was 25.309 µm with an average S/N ratio of −27.891 dB, the average DD value was 0.0773 mm with an average S/N ratio of 22.493 dB, and the average CD value was 0.0288 mm with an average S/N ratio of 31.154 dB.

**Figure 8:**
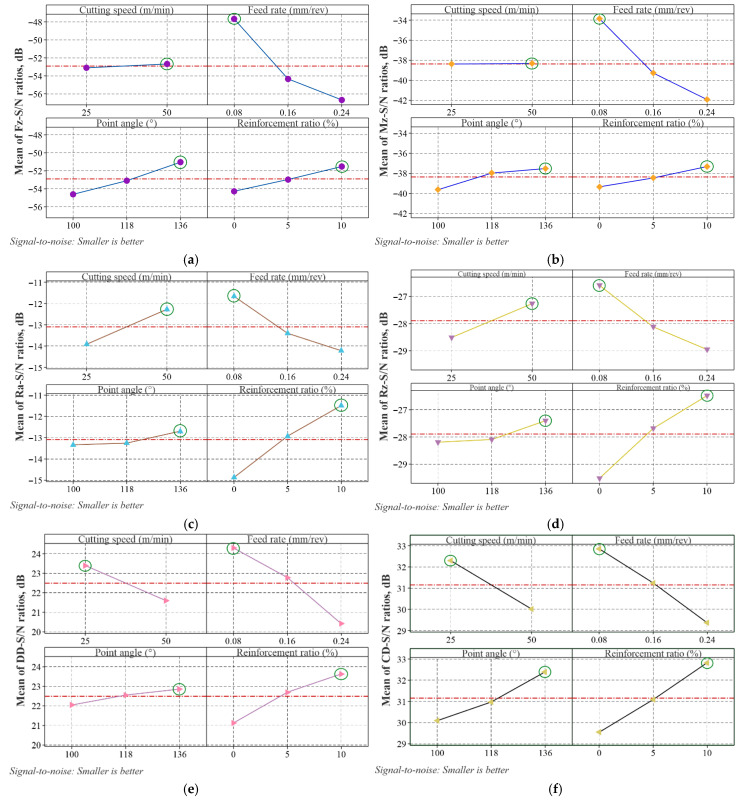
Main effect plots for S/N ratios; (**a**) Fz, (**b**) Mz, (**c**) Ra, (**d**) Rz, (**e**) DD, (**f**) CD.

The main effect plots of the S/N ratios for the responses are shown in Figure 8. The optimal experimental condition for each response consists of the levels of control factors that have the highest S/N ratios. When the figure is examined, the optimal levels of the control factors for Fz, Mz, Ra, and Rz are Vc: 50 m/min, f: 0.08 mm/rev, PA: 136°, and RR: 10%. For DD and CD, only the Vc changes to 25 m/min, while the levels of the other control factors remain the same. Thus, using the S/N analysis of the Taguchi method, the optimal experimental conditions for each response have been determined.

#### 3.3.2. Evaluation of Main Effect Graphs for Responses

**Figure 9:**
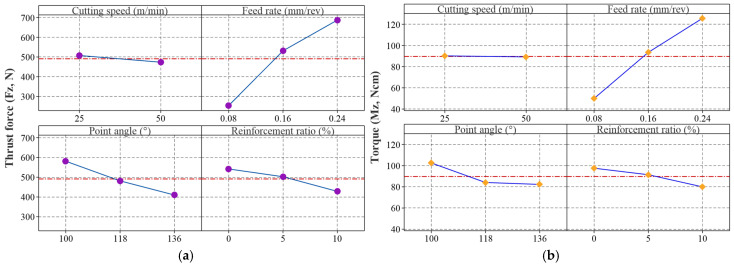

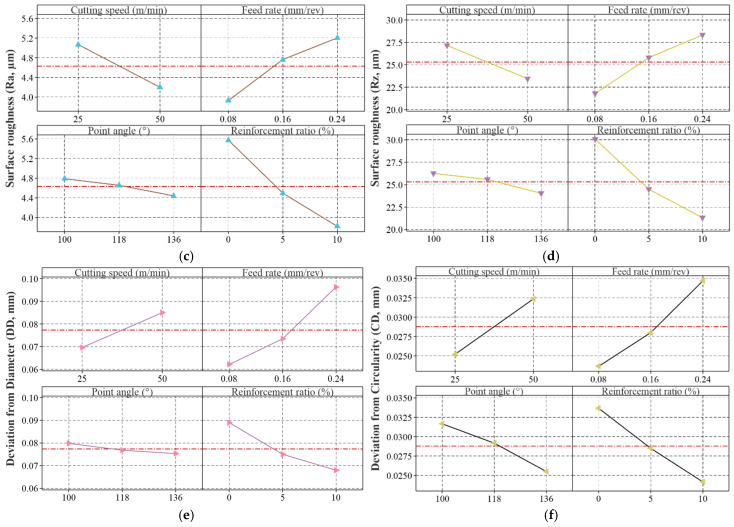
Main effect plots for the responses; (**a**) Fz, (**b**) Mz, (**c**) Ra, (**d**) Rz, (**e**) DD, (**f**) CD.

**Figure 10:**
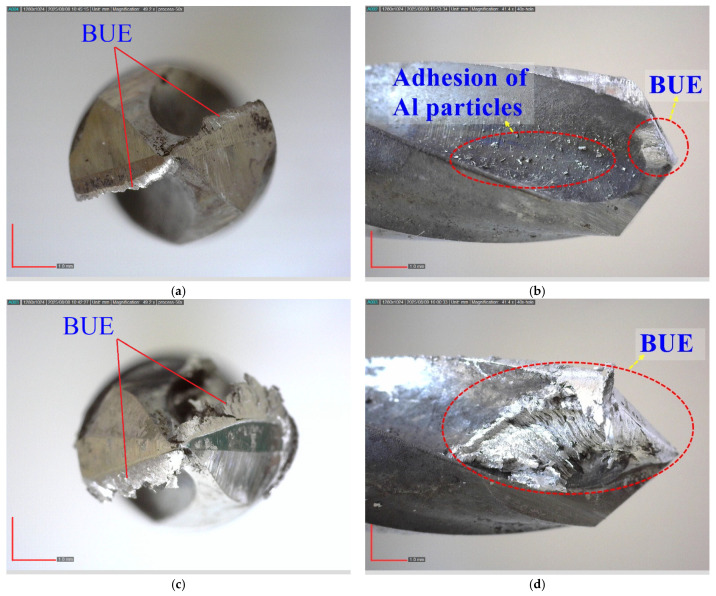
Images of drills after drilling; 3rd experimental condition (**a**) Top view, (**b**) Side view; 7th experimental condition (**c**) Top view, (**d**) Side view.

**Figure 11:**
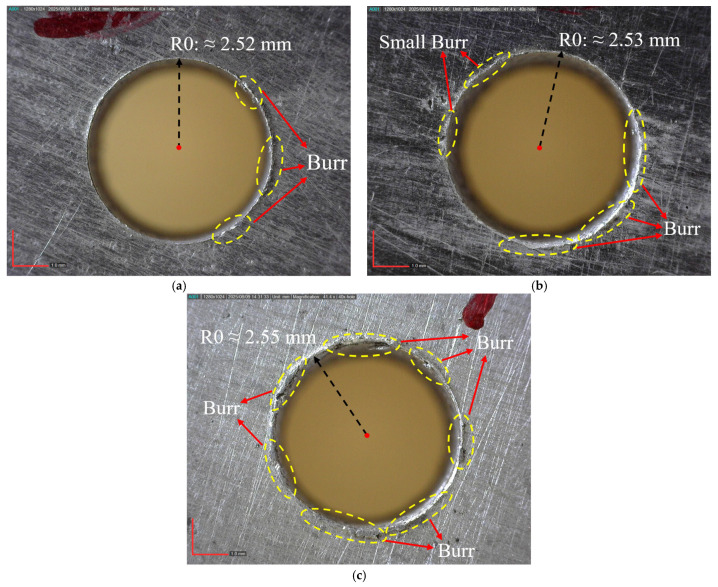
Hole images obtained according to drilling conditions; (**a**) 3rd, (**b**) 5th and (**c**) 9th (experimental conditions).

Figure 9 shows the main effect plots of the hole quality indicators. As seen in Figure 9a,b, both Fz and Mz increase with higher feed rates due to the greater material removal per revolution. Increasing the PA from 100° to 136° significantly reduces Fz, while the reduction in Mz is more moderate. This indicates that larger PA promote efficient cutting by focusing the force at the tip and aiding chip fragmentation. As demonstrated by Clauß et al. [56], drilling AMHCs presents unique challenges because of the presence of hard reinforcement particles, which can lead to increased tool wear, high Fz, and poor surface finish. The PA of the drill bit is a significant factor influencing the drilling method and hence cutting efficiency, chip formation, and surface quality. The results of the present study support these findings, confirming that careful choosing of the PA can reduce the drawbacks during drilling and hence improve the hole quality. Further, increasing the reinforcement by 10% decreases the Fz and Mz. This trend is attributed not only to the increased brittleness but also to the reduction in Al-matrix adhesion and BUE formation on the tool surface, as observed in Figure 10 and Figure 11. The cleaner cutting edges and reduced smearing tendency with higher reinforcement led to lower thrust and torque values, with the lowest forces recorded in the 10% SiC specimens (e.g., Fz decreased from 400 N at RR = 0% to 150 N at RR = 10% under identical feed and point angle; Table 3) [14].

A similar trend is observed in Ra and Rz (Figure 9c,d). These increase with feed rate but decrease with larger PA and higher RR, suggesting improved cutting stability and a stiffer composite structure. Higher Vc also enhance surface quality, likely by reducing built-up edge formation and promoting thermal softening of the matrix. As demonstrated by Saini et al., higher Vc improve surface quality in drilling AMHCs by minimizing built-up edge and facilitating matrix softening [57]. This is supported by multiple studies indicating that optimized cutting parameters—particularly increased Vc combined with suitable tool materials—contribute to smoother surfaces and reduced surface irregularities in composite drilling operations. Figure 9e,f, showing DD and CD, indicate that dimensional accuracy deteriorates at higher feed rates due to increased tool deflection. However, both deviations decrease with increasing PA and RR, as a sharper tool and stiffer material help maintain hole geometry. Interestingly, while a high Vc improves surface finish, it slightly worsens DD and CD, possibly due to thermal effects. Overall, low feed rate (0.08 mm/rev), high PA (136°), and 10% reinforcement offer optimal conditions for minimizing Fz, Mz improving surface finish (Ra, Rz), and ensuring dimensional accuracy (DD, CD). Although higher Vc (50 m/min) benefits surface quality (Ra, Rz), a lower Vc (25 m/min) is more favourable for geometric precision (DD, CD).

Figure 10a,b present the top and side views of the drill bits after drilling under the 3rd experimental condition (Vc: 25 m/min, f: 0.08 mm/rev, PA: 136°, SiC-RR: 10%). The low feed rate and large PA led to the formation of thinner chips in the cutting zone, thereby limiting the accumulation of BUE and preserving the cutting-edge geometry. This, in turn, improved both the hole surface quality and geometric accuracy. In contrast, Figure 10c,d show the drill bits from the 7th experimental condition (Vc: 25 m/min, f: 0.24 mm/rev, PA: 100°, SiC-RR: 5%), where the high feed rate and small PA resulted in thicker chips and the formation of dense BUE layers on the cutting edge. Combined with the high adhesion tendency of the pure Al matrix, this led to deterioration of the drill geometry, obstruction of chip flow, increased cutting forces, and a significant reduction in hole quality.

The hole images in Figure 11 clearly show the effects of different drilling parameters on surface and edge quality. Figure 11a was obtained under the conditions of the third experiment (Vc: 25 m/min, f: 0.08 mm/rev, PA: 136°, RR: 10% SiC) and shows distinct edge lines, minimal burr formation, and extremely low deviation from circularity. The low feed rate and large PA ensured controlled chip removal; the hard and reinforced structure prevented edge crushing. The hole radius was measured to be approximately 2.52 mm. Figure 11b was obtained under the conditions of the 5th experiment (Vc: 25 m/min, f: 0.16 mm/rev, PA: 118°, RR: 5% SiC) and shows burrs and surface ripples in places on the edges; this is because the increased feed rate increases the load on the tool and micro-vibrations. The hole radius here was measured to be approximately 2.53 mm. Figure 11c reflects the result of the 9th experiment (Vc: 25 m/min, f: 0.24 mm/rev, PA: 136°, RR: 0% SiC) and shows the lowest quality; High feed rates and the absence of reinforcement increased plastic deformation and adhesion tendencies in the cutting zone, leading to significant burr formation, edge rounding, and loss of circularity. The hole radius was measured as 2.55 mm. Hole quality is clearly defined in the order a > b > c.

#### 3.3.3. ANOVA Results

**Figure 12:**
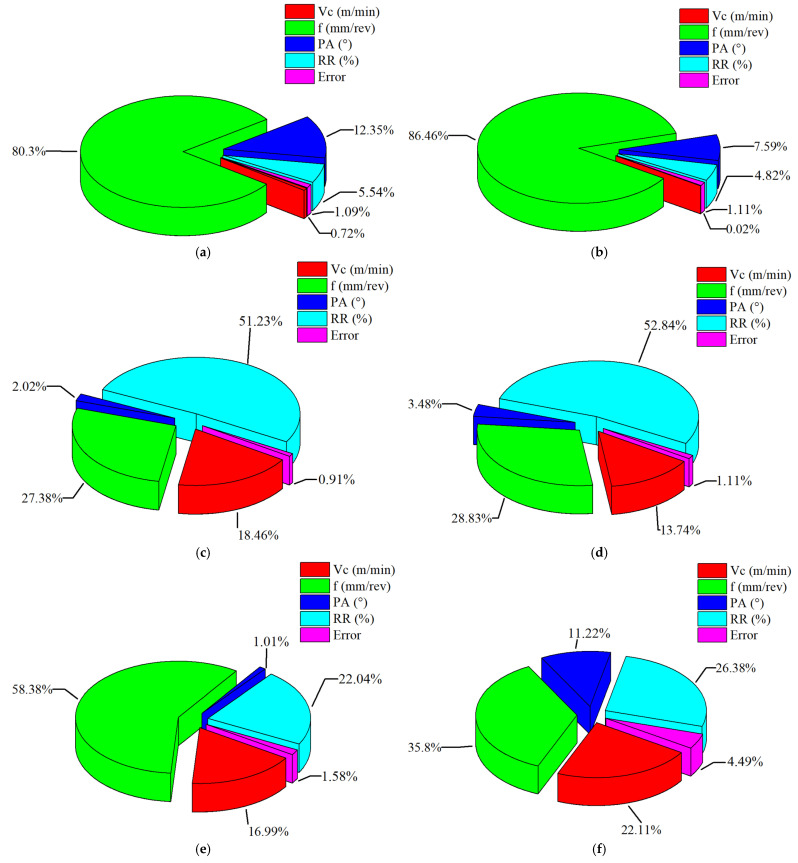
Percentage contribution ratios of control factors based on ANOVA results; (**a**) Fz, (**b**) Mz, (**c**) Ra, (**d**) Rz, (**e**) DD, (**f**) CD.

The ANOVA results for the hole quality responses obtained after the drilling of AMHCs are presented in Table 4, where the statistical significance of each control parameter—Vc, f, PA, and RR—on the respective quality characteristics is evaluated. Additionally, the percentage contribution ratios (PCR%) of these control factors, as determined by the ANOVA, are illustrated in detail in Figure 12. For Fz, the most dominant factor was identified as the feed rate, contributing 80.30%, followed by the PA (12.35%) and RR (5.54%). Although the contribution of Vc was relatively low (0.72%), it was still statistically significant at the *p* < 0.05 level. A comparable pattern was noted for Mz, where feed rate again exhibited the highest contribution (86.47%), followed by PA (7.59%) and RR (4.82%), while the effect of Vc was statistically insignificant (*p* = 0.662). For Ra, the RR had the highest impact with a contribution of 51.22%, followed by feed rate (27.38%) and Vc (18.46%), whereas the contribution of PA (2.02%) was minimal. Likewise, for Rz, RR was again the most influential factor (52.84%), while feed rate (28.83%) and Vc (13.74%) also had statistically significant effects. Regarding the geometric accuracy indicators, the most significant factor affecting DD was feed rate (58.37%), followed by RR (22.04%), while PA showed an insignificant effect (*p* = 0.085).

For CD, feed rate (35.80%), RR (26.38%), and Vc (22.11%) all exhibited notable influences. Overall, the ANOVA results evidently illustrate that feed rate is the most dominant and statistically crucial factor across all quality responses. While the RR significantly influences surface and geometric characteristics, the PA has a more pronounced effect on Fz and Mz. Vc, on the other hand, has limited but statistically meaningful effects on Ra and geometric accuracy. The high values of R^2^ (≥95.5%) and adjusted R^2^ observed for all responses confirm the strong explanatory power of the regression models, thereby validating the reliability of the analyses. This comprehensive ANOVA evaluation provides a detailed understanding of the influence of each factor and forms a solid basis for the subsequent multi-response optimization stage.

#### 3.3.4. Regression Analysis

To model and quantify the relationships between the drilling parameters and the six hole quality indicators, multiple linear regression analyses were performed. The resulting regression equations for Fz, Mz, Ra, Rz, DD, and CD are provided in Equations (21)–(26), respectively. These equations express each response variable as a linear function of the input parameters. The regression model for Fz exhibited a strong fit, with an R^2^ of 96.57%, indicating that over 96% of the variation in Fz can be explained by the input variables [58]. Among the coefficients, the feed rate had the most dominant positive influence on Fz, whereas PA and RR had negative coefficients, implying that increasing PA and RR reduces Fz. Similarly, the model for Mz showed an R^2^ value of 96.72%, again suggesting excellent model reliability. Feed rate remained the most influential factor, while RR had a notable but lesser negative effect. For surface quality indicators, the regression equations of Ra and Rz revealed that both responses are significantly affected by all four control parameters. The model for Ra achieved the highest R^2^ value among all responses (97.33%), and Rz also demonstrated a robust model fit with an R^2^ of 96.94%. Notably, feed rate showed a strong positive correlation with both Ra and Rz, indicating rougher surfaces at higher feed rates, whereas increasing RR and PA improved surface quality by reducing roughness values. In terms of dimensional accuracy, the regression models for DD and CD yielded R^2^ values of 95.47% and 94.78%, respectively, confirming good predictive power. The feed rate was again the primary factor increasing both DD and CD. In contrast, the PA and RR exhibited negative coefficients, suggesting that larger drill PA and higher RR contribute to more accurate hole geometry. Overall, the high R^2^, adjusted R^2^, and predicted R^2^ values for all six models validate the reliability and generalizability of the regression equations. These models offer valuable insights into the quantitative effects of drilling parameters and can be used as predictive tools to optimize the drilling process of AMHCs.

(21) $$Fz\left(N\right)=727.2\;-1.360Vc+2704f-4.755PA-11.33RR$$

R^2^: 96.57%, R^2^ (adj): 95.52%, R^2^ (pred): 93.62%

(22) $$Mz\left(Ncm\right)=91-0.040Vc+472.9f-0.565PA-1.767RR$$

R^2^: 96.72%, R^2^ (adj): 95.71%, R^2^ (pred): 93.67%

(23) $$Ra\left(µm\right)=6.679-0.03474Vc+7.971f-0.00969PA-0.1756RR$$

R^2^: 97.33%, R^2^ (adj): 96.51%, R^2^ (pred): 95.16%

(24) $$Rz\left(µm\right)=36.04-0.1481Vc+40.71f-0.0618PA-0.8785RR$$

R^2^: 96.94%, R^2^ (adj): 96%, R^2^ (pred): 94.31%

(25) $$DD\left(mm\right)=0.04542+0.000613Vc+0.2135f-0.000125PA-0.0021RR$$

R^2^: 95.47%, R^2^ (adj): 94.07%, R^2^ (pred): 91.36%

(26) $$CD\left(mm\right)=0.03207+0.000284Vc+0.06875f-0.000171PA-0.00095RR$$

R^2^: 94.78, R^2^ (adj): 93.17%, R^2^ (pred): 88.95%

#### 3.3.5. Calculation of Criterion Weights Using the Entropy Method

In the drilling process of AMHCs, the optimal drilling parameters that minimize the performance criteria—namely Fz, Mz, Ra, Rz, DD, and CD—were determined using a Taguchi-based MCDM method. In MCDM methods, the weighting of performance criteria is of critical importance, as the weights significantly influence the ranking of alternatives. In this study, the Entropy Method, one of the objective weighting approaches, was employed to assign weights to the performance criteria. The weights were calculated directly based on the raw criterion data. First, a decision matrix was constructed using performance data obtained from the Taguchi L_18_ experimental design. The performance criterion values in the decision matrix were normalized using Equation (8). The entropy coefficients were calculated utilizing Equations (9) and (10), the degree of diversification was established using Equation (11), the weights of the performance criteria were calculated using Equation (12). Table 5 presents the weight values of the performance criteria as determined by the Entropy Method. As a result of the entropy-based calculations, the weights of the performance criteria were obtained as follows: 33.23% for Fz, 26.70% for Mz, 8.92% for Ra, 7.25% for Rz, 10.71% for DD, and 13.19% for CD.

#### 3.3.6. Taguchi-Based Entropy–CoCoSo Method

The optimal drilling condition that minimizes all performance criteria in the drilling of AMHCs was determined using the Taguchi-based Entropy–CoCoSo method. A decision matrix was constructed for the multiple performance criteria obtained according to the Taguchi L_18_ experimental design. Since the objective was to minimize all performance criteria, normalization was performed using Equation (14), which is applicable to cost-based criteria. The *Si* and *Pi* values were calculated using Equations (15) and (16), respectively. Here, $w_{j}$, represents the weight of each criterion, which was determined using the Entropy method. By substituting the $S_{i}$ and $P_{i}$ values into Equations (17)–(19), the relative performance scores of the decision alternatives ($k_{ia}$, $k_{ib}$ and $k_{ic}$) were calculated. In Equation (19), the λ value was set to 0.5. Subsequently, the performance indicator of the decision alternatives ($k_{i}$) was calculated based on the relative performance scores using Equation (20). Then, the S/N ratios of the $k_{i}$ values were determined using Equation (5), in accordance with the “larger-is-better” objective function. Table 6 presents the calculated relative performance scores $k_{i}$ values, and their corresponding S/N ratios.

**Figure 13:**
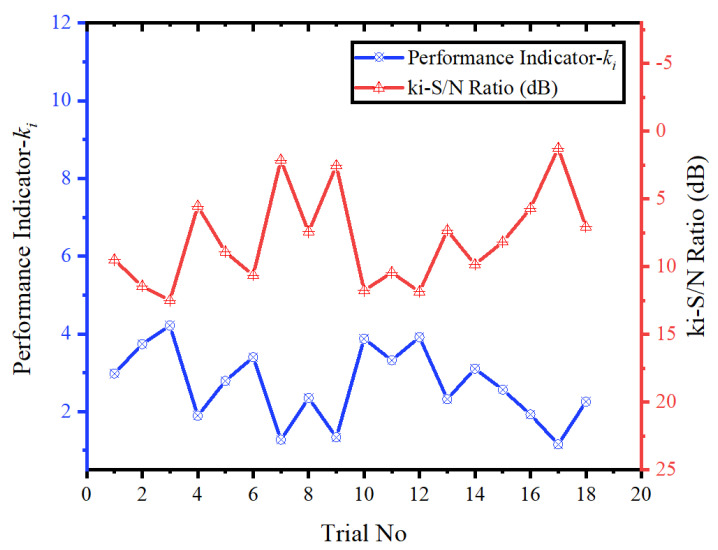
Graph of *k_i_* values and S/N ratios by experiment number.

The graph of the calculated $k_{i}$ values and their corresponding S/N ratios based on experiment numbers is shown in Figure 13. In this graph, the highest $k_{i}$ and $k_{i}$-S/N ratio indicate the best experimental condition among the alternatives. As observed in Figure 13, the highest $k_{i}$ and $k_{i}$-S/N ratio values were obtained for Experiment No. 3, whereas the lowest values were observed for Experiment No. 17. Accordingly, the experimental combination that exhibits the best performance in terms of multi-response quality characteristics corresponds to Vc: 25 m/min, f: 0.08 mm/rev, PA: 136°, and RR: 10%. On the other hand, the worst-performing experimental condition corresponds to Vc: 50 m/min, f: 0.24 mm/rev, PA: 118°, and RR: 0%.

**Figure 14:**
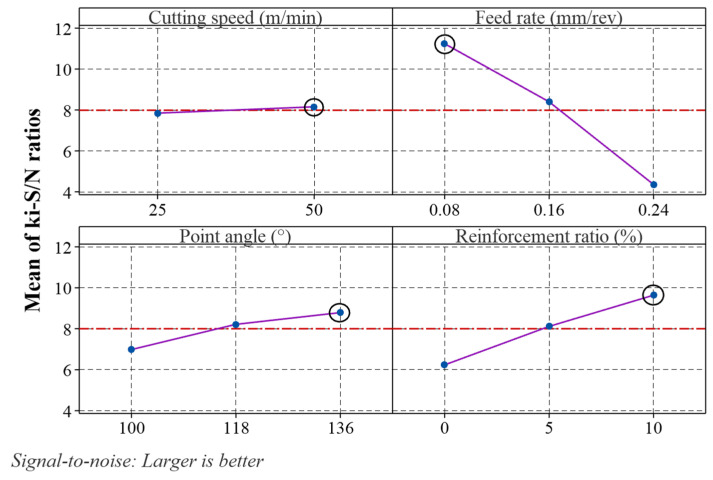
Main effects plot for *k_i_*-S/N ratio.

In the Taguchi-based Entropy–CoCoSo method, the $k_{i}$-S/N ratio graph was used to determine the optimal experimental condition (Figure 14). The levels with the highest $k_{i}$-S/N ratios in the graph represent the optimal experimental conditions. According to the $k_{i}$-S/N ratio graph, the optimal experimental combination for achieving the highest $k_{i}$ value corresponds to Vc: 50 m/min, f: 0.08 mm/rev, PA: 136°, and RR: 10%.

**Figure 15:**
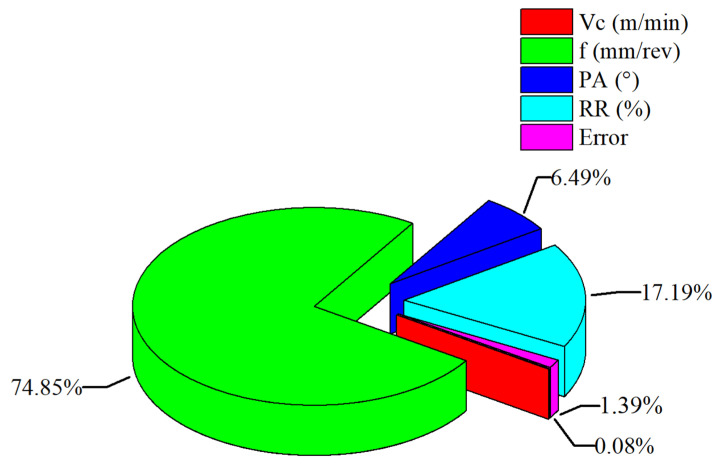
Percentage contribution of control factors to *k_i_* based on ANOVA results.

The ANOVA results for $k_{i}$ are given in Table 7. According to, the contribution ratios of the control factors to $k_{i}$ were found to be 74.85% for feed rate, 17.19% for RR, 6.49% for PA, and 0.08% for Vc. Additionally, the low error value (1.39%) indicates that the experimental data are statistically crucial. The results clearly show that feed rate is the most significant parameter on $k_{i}$. Although RR and PA are also effective parameters, Vc was found to have a negligible effect. The coefficients of determination for the ANOVA model of $k_{i}$ were obtained as follows: R^2^: 98.61%, adjusted R^2^: 97.64% and predicted R^2^: 95.51%. Figure 15 illustrates the percentage contribution of each control factor to $k_{i}$.

Regression analysis was used to mathematically express the relationship between $k_{i}$ and the control factors. Equation (27) presents the first-order regression model developed $k_{i}$.

(27) $$k_{i}=2.239+0.00203Vc-12.225f+0.01579PA+0.09363RR$$

The coefficients of determination for the mathematical model were obtained as follows: R^2^: 98.40%, adjusted R^2^: 97.91%, predicted R^2^: 96.83%. In this study, the high R^2^ values of the developed model for $k_{i}$ indicate that the model has a strong predictive capability. While several MCDM methods such as GRA, TOPSIS, and VIKOR are largely used in drilling optimization, combining Entropy with CoCoSo within the Taguchi framework lends an original balance between objective weighting and compromise-based ranking. Even though the parameter ranges in this investigation were set to represent industrial practical drilling conditions, the method is generalizable to larger ranges and to other machining processes. Moreover, the high R^2^ values found and the confirmation tests within the 95% confidence interval show that these models are not overfitted and considered reasonably good for predictive and optimization uses.

#### 3.3.7. Validation Experiments and Calculation of Confidence Intervals

According to the Taguchi-based Entropy–CoCoSo method, the optimal experimental combination was determined as (Vc_2_ f_1_ PA_3_ RR_3_). Verification experiments were conducted with this combination to test whether the predicted improvement was achieved. Through the optimization process, the estimated kᵢ value was calculated by considering the drilling parameters: Vc: 50 m/min, f: 0.08 mm/rev, PA: 136°, and RR: 10%. In estimating the optimal $k_{i}$ value, the response table for the average $k_{i}$ values given in Table 8 and Equation (28) were used.

**Table 8:** Response table for the average *k_i_*.

Level	Vc (m/min)	f (mm/rev)	PA (°)	RR (%)
1	2.664	3.673 *	2.379	2.209
2	2.715 *	2.678	2.743	2.715
3		1.717	2.947 *	3.145 *
Delta	0.051	1.956	0.568	0.936
Total average value of the $k_{i}$ = 2.690				

* Optimal levels of control factors.

(28) $${k_{i}}_{opt}=\left({Vc}_{2}-T_{k_{i}}\right)+\left(f_{1}-T_{k_{i}}\right)+\left({PA}_{3}-T_{k_{i}}\right)+\left({RR}_{3}-T_{k_{i}}\right)+T_{k_{i}}$$

The average of the $k_{i}$ values calculated using the Taguchi-based Entropy–CoCoSo method is denoted as $T_{k_{i}}$, while the predicted optimal value is denoted as ${k_{i}}_{opt}$. The values of $T_{k_{i}}$ and ${k_{i}}_{opt}$ were calculated as 2.690 and 4.41, respectively. Since the optimal experimental combination (Vc_2_ f_1_ PA_3_ RR_3_) was not included in the Taguchi L_18_ design, verification experiments were conducted under this condition, results are demonstrated in Table 9. The $k_{i}$ value obtained from the verification experiment was 4.206, while the predicted $k_{i}$ value according to the Taguchi method was 4.41. Furthermore, when comparing the results of the initial parameter set and the verification experiment, an improvement of 1.224 in $k_{i}$ was observed, corresponding to a 41.05% improvement rate. These results demonstrate that the Taguchi-based Entropy–CoCoSo method can be effectively applied for the optimization of $k_{i}$.

To confirm the reliability of the optimization process, the confidence interval (CI) of the predicted $k_{i}$ value was calculated using Equations (29) and (30), based on various parameter values provided in Table 10 [59,60]. The resulting confidence interval was determined as ${CI}_{k_{i}}$ = ±0.287. The confidence interval for the predicted k_i_ value at a 95% confidence level was then calculated using Equation (31).

(29) $$CI=\sqrt{F_{\ddot{\alpha }:1,{Sd}_{e}}{\bar{V}}_{e}\left(\frac{1}{n_{eff}}+\frac{1}{\bar{r}}\right)}$$

(30) $$n_{eff}=\frac{n}{1+T_{dof}}$$

(31) $$\begin{array}{cc} \left[{k_{i}}_{opt}-{CI}_{k_{i}}\right]< & {k_{i}}_{exp}<\left[{k_{i}}_{opt}+{CI}_{k_{i}}\right]\to \left[4.41-0.287\right]<4.206\\ & <\left[4.41+0.287\right]=4.123<4.206<4.697 \end{array}$$

The results of the confirmation experiments (${k_{i}}_{exp}$) were found to fall within the confidence interval limits (${CI}_{k_{i}}$). This indicates that the optimization of $k_{i}$ using the Taguchi-based CoCoSo method was successfully implemented at a 95% confidence level and a 5% significance level.

## 4. Conclusions

This study is a huge investigation into drilling characteristics on aluminum matrix hybrid composites (AMHCs) reinforced with boron carbide (B_4_C) and another different amount of silicon carbide (SiC: 0%, 5%, and 10%) fabricated via the direct hot-pressing technique. The study aims to seek optimal drilling parameters through single- and multi-response strategies, wherein the Taguchi method and an innovative Taguchi-based Entropy–CoCoSo hybrid approach are used. Six critical hole quality indicators, namely, thrust force (Fz), torque (Mz), average surface roughness (Ra), maximum surface roughness (Rz), diameter deviation (DD), and circularity deviation (CD) were analyzed in detail. A few of the unrevealed views that this work brought forth towards progressing the machinability and optimization methodology concerning AMHC might be worthy of future study.

From a materials perspective, increasing the SiC content significantly altered the composite’s mechanical properties. The hardness improved from 65.5 HB in the 0% SiC condition to 76.9 HB in the 10% SiC condition, driven by the inherent hardness of SiC particles and the load-bearing capacity they impart to the matrix. However, this improvement came at the cost of transverse rupture strength (TRS), which decreased from 140 MPa to 71 MPa as porosity increased with higher reinforcement. SEM/EDX analyses confirmed a generally homogeneous distribution of B_4_C and SiC particles, although some localized agglomeration was observed. The microstructural findings align with the mechanical test results, highlighting the typical trade-off between strength and hardness in ceramic-reinforced metal matrices.

In the drilling experiments, the single-response Taguchi analysis revealed that optimal parameters for minimizing Fz, Mz, Ra, and Rz consisted of Vc: 50 m/min, f: 0.08 mm/rev, PA: 136°, and RR: 10% SiC. For DD and CD, the same parameters were optimal except for Vc, where 25 m/min produced superior geometric accuracy. These results underline the sensitivity of different quality characteristics to specific machining conditions, emphasizing that a universal parameter set may not always be optimal across all metrics. ANOVA results consistently identified feed rate as the most significant factor for all quality indicators, contributing up to 86.47% for Mz and 80.30% for Fz. The RR was the dominant factor for surface quality indicators (Ra and Rz) and exerted a substantial influence on geometric accuracy metrics (DD and CD). PA significantly affected Fz and Mz, while Vc had a moderate but statistically significant impact on Ra, Rz and DD, CD. The multiple linear regression models achieved high coefficients of determination (R^2^ ≥ 94.78%), confirming their strong predictive power and enabling quantitative estimations of hole quality based on the chosen parameters.

For multi-response optimization, the Entropy method assigned the highest weights to Fz (33.23%) and Mz (26.70%), emphasizing the importance of reducing cutting loads for tool life and process stability. CoCoSo ranking showed that Vc: 25 m/min, f: 0.08 mm/rev, PA: 136°, and RR: 10% SiC yielded the best run, while the Taguchi–Entropy–CoCoSo integration identified Vc: 50 m/min, f: 0.08 mm/rev, PA: 136°, and RR: 10% SiC as the most balanced setting. Confirmation experiments verified this result with a 41.05% improvement in the overall index (kᵢ), and the experimental value (4.206) fell within the 95% confidence interval, confirming robustness. The novelty of this work lies in the integrated evaluation of six-hole quality indicators and in demonstrating that the Taguchi-based Entropy–CoCoSo method provides a reliable framework for multi-criteria drilling optimization beyond conventional single-response or unweighted approaches. Notably, while the CoCoSo run-wise ranking identified Vc = 25 m/min as the best-performing tested run, the Taguchi–Entropy–CoCoSo main-effects analysis and subsequent confirmation tests validated Vc = 50 m/min as the most balanced multi-response optimum for production. Nevertheless, if geometric accuracy (DD and CD) is prioritized over overall surface integrity, operating at 25 m/min can be considered as a context-specific alternative.

From a practical viewpoint, this study shows that low feed rate, large point angle, and high ceramic reinforcement ratio improve surface integrity and dimensional accuracy in AMHC drilling, while cutting speed must be balanced between surface finish and geometric precision. Although the experiments were limited to uncoated HSS drills and did not track tool wear progression, the findings highlight important directions for future research, including the use of different tool geometries, environments (dry or lubricated), advanced tool materials, and high-performance cooling/lubrication methods. Moreover, extending the methodology to incorporate additional criteria such as energy consumption, material removal rate, and burr formation would allow for more holistic optimization. While the Taguchi-based Entropy–CoCoSo method proved highly effective, exploring alternative weighting methods and conducting sensitivity analyses could further strengthen its generality. Overall, the regression models and optimization framework developed here provide a robust and flexible tool that can be applied to different AMHC systems and machining operations, offering valuable insights into the machinability of B_4_C–SiC reinforced composites.

## Figures and Tables

**Table 1:** Drilling parameters and levels.

Control Factors	Symbol	Levels		
		1	2	3
Cutting speed (m/min)	Vc	25	50	–
Feed rate (mm/rev)	f	0.08	0.16	0.24
Point angle (°)	PA	100	118	136
SiC reinforcement ratio (%)	RR	0	5	10

**Table 2:** Theoretical, experimental, and relative density values.

Composite Material	Theoretical Density (g/cm^3^)	Experimental Density (g/cm^3^)	Relative Density (%)
Al/5B_4_C	2.691	2.638	98.02
Al/5B_4_C/5SiC	2.717	2.633	96.92
Al/5B_4_C/10SiC	2.742	2.580	94.10

**Table 3:** Measured responses and computed S/N ratios.

Trial No	Vc (m/min)	f (mm/rev)	PA (°)	RR (%)	Fz (N)	Fz-S/N (dB)	Mz (Ncm)	Mz-S/N (dB)	Ra (µm)	Ra-S/N (dB)	Rz (µm)	Rz-S/N (dB)	DD (mm)	DD-S/N (dB)	CD (mm)	CD-S/N (dB)
1	25	0.08	100	0	400	−52.041	67	−36.521	5.463	−14.749	29.188	−29.304	0.072	22.853	0.030	30.458
2	25	0.08	118	5	260	−48.299	48	−33.625	4.128	−12.315	22.646	−27.100	0.053	25.514	0.021	33.556
3	25	0.08	136	10	150	−43.522	40	−32.041	3.366	−10.542	18.046	−25.128	0.043	27.331	0.013	37.721
4	25	0.16	100	0	685	−56.714	115	−41.214	6.222	−15.879	33.745	−30.564	0.082	21.724	0.033	29.630
5	25	0.16	118	5	520	−54.320	89	−38.988	5.186	−14.297	26.709	−28.533	0.063	24.013	0.025	32.041
6	25	0.16	136	10	385	−51.709	71	−37.025	4.094	−12.243	22.446	−27.023	0.056	25.036	0.018	34.895
7	25	0.24	100	5	837	−58.455	145	−43.227	5.594	−14.954	30.104	−29.572	0.085	21.412	0.032	29.897
8	25	0.24	118	10	653	−56.298	110	−40.828	5.034	−14.038	27.229	−28.701	0.076	22.384	0.025	32.041
9	25	0.24	136	0	683	−56.688	127	−42.076	6.494	−16.250	34.333	−30.714	0.097	20.265	0.030	30.458
10	50	0.08	100	10	237	−47.495	48	−33.625	2.822	−9.011	17.154	−24.687	0.061	24.293	0.023	32.765
11	50	0.08	118	0	295	−49.396	52	−34.320	4.566	−13.191	25.854	−28.251	0.078	22.158	0.032	29.897
12	50	0.08	136	5	185	−45.343	45	−33.064	3.218	−10.152	17.946	−25.079	0.066	23.609	0.023	32.765
13	50	0.16	100	5	646	−56.205	109	−40.749	4.494	−13.053	24.819	−27.896	0.080	21.938	0.033	29.630
14	50	0.16	118	10	464	−53.330	79	−37.953	3.446	−10.746	20.383	−26.185	0.073	22.734	0.027	31.373
15	50	0.16	136	0	490	−53.804	98	−39.825	5.126	−14.196	26.709	−28.533	0.087	21.210	0.032	29.897
16	50	0.24	100	10	683	−56.688	132	−42.411	4.153	−12.367	22.646	−27.100	0.099	20.087	0.039	28.179
17	50	0.24	118	0	699	−56.890	127	−42.076	5.582	−14.936	30.786	−29.767	0.118	18.562	0.045	26.936
18	50	0.24	136	5	568	−55.087	113	−41.062	4.358	−12.786	24.819	−27.896	0.103	19.743	0.037	28.636
Average:					491.111	−52.905	89.722	−38.368	4.630	−13.095	25.309	−27.891	0.0773	22.493	0.0288	31.154
The greatest value:					837	−43.522	145	−32.041	6.494	−9.011	34.333	−24.687	0.118	27.331	0.045	37.721
The smallest value:					150	−58.455	40	−43.227	2.822	−16.25	17.154	−30.714	0.043	18.562	0.013	26.936

**Table 4:** ANOVA results for the responses.

Factors	DF	Seq SS	Adj MS	F-Value	*p*-Value	PCR %
Fz (N)						
Vc (m/min)	1	5202	5202	6.64	0.028	0.72
f (mm/rev)	2	576,404	288,202	368.08	*p* < 0.001	80.30
PA (°)	2	88,669	44,334	56.62	*p* < 0.001	12.35
RR (%)	2	39,735	19,868	25.37	*p* < 0.001	5.54
Error	10	7830	783			1.09
Total	17	717,840				100
R^2^: 98.91%, R^2^ (adj): 98.15%, R^2^ (pred): 96.47%						
Mz (Ncm)						
Vc (m/min)	1	4.5	4.50	0.20	0.662	0.02
f (mm/rev)	2	17,304.8	8652.39	390.73	*p* < 0.001	86.47
PA (°)	2	1518.1	759.06	34.28	*p* < 0.001	7.59
RR (%)	2	964.8	482.39	21.78	*p* < 0.001	4.82
Error	10	221.4	22.14			1.11
Total	17	20,013.6				100
R^2^: 98.89%, R^2^ (adj): 98.12%, R^2^ (pred): 96.42%						
Ra (µm)						
Vc (m/min)	1	3.3939	3.39388	202.26	*p* < 0.001	18.46
f (mm/rev)	2	5.0339	2.51694	150.00	*p* < 0.001	27.38
PA (°)	2	0.3711	0.18555	11.06	0.003	2.02
RR (%)	2	9.4157	4.70786	280.57	*p* < 0.001	51.22
Error	10	0.1678	0.01678			0.91
Total	17	18.3824				100
R^2^: 99.09%, R^2^ (adj): 98.45%, R^2^ (pred): 97.04%						
Rz (µm)						
Vc (m/min)	1	61.716	61.716	123.90	*p* < 0.001	13.74
f (mm/rev)	2	129.476	64.738	129.97	*p* < 0.001	28.83
PA (°)	2	15.636	7.818	15.69	0.001	3.48
RR (%)	2	237.324	118.662	238.22	*p* < 0.001	52.84
Error	10	4.981	0.498			1.11
Total	17	449.133				100
R^2^: 98.89%, R^2^ (adj): 98.11%, R^2^ (pred): 96.41%						
DD (mm)						
Vc (m/min)	1	0.001058	0.001058	107.23	*p* < 0.001	16.99
f (mm/rev)	2	0.003634	0.001817	184.17	*p* < 0.001	58.37
PA (°)	2	0.000063	0.000032	3.19	0.085	1.01
RR (%)	2	0.001372	0.000686	69.53	*p* < 0.001	22.04
Error	10	0.000099	0.000010			1.58
Total	17	0.006226				100
R^2^: 98.42%, R^2^ (adj): 97.31%, R^2^ (pred): 94.87%						
CD (mm)						
Vc (m/min)	1	0.000228	0.000228	49.23	*p* < 0.001	22.11
f (mm/rev)	2	0.000368	0.000184	39.86	*p* < 0.001	35.80
PA (°)	2	0.000115	0.000058	12.49	0.002	11.22
RR (%)	2	0.000271	0.000136	29.36	*p* < 0.001	26.38
Error	10	0.000046	0.000005			4.49
Total	17	0.001029				100
R^2^: 95.51%, R^2^ (adj): 92.36%, R^2^ (pred): 85.45%						

**Table 5:** Weight values of performance criteria calculated according to the Entropy method.

Trial No	Fz	Mz	Ra	Rz	DD	CD
1	−0.1401	−0.1320	−0.1786	−0.1761	−0.1532	−0.1650
2	−0.1037	−0.1045	−0.1488	−0.1492	−0.1244	−0.1300
3	−0.0692	−0.0916	−0.1296	−0.1279	−0.1074	−0.0925
4	−0.1982	−0.1881	−0.1937	−0.1928	−0.1668	−0.1754
5	−0.1667	−0.1597	−0.1728	−0.1663	−0.1401	−0.1463
6	−0.1365	−0.1374	−0.1480	−0.1483	−0.1293	−0.1167
7	−0.2232	−0.2164	−0.1813	−0.1795	−0.1707	−0.1720
8	−0.1925	−0.1830	−0.1695	−0.1684	−0.1588	−0.1463
9	−0.1978	−0.2000	−0.1989	−0.1948	−0.1856	−0.1650
10	−0.0970	−0.1045	−0.1146	−0.1235	−0.1371	−0.1383
11	−0.1135	−0.1106	−0.1591	−0.1628	−0.1615	−0.1720
12	−0.0809	−0.0998	−0.1256	−0.1274	−0.1446	−0.1383
13	−0.1912	−0.1819	−0.1575	−0.1585	−0.1642	−0.1754
14	−0.1547	−0.1476	−0.1317	−0.1390	−0.1546	−0.1540
15	−0.1603	−0.1700	−0.1715	−0.1663	−0.1733	−0.1720
16	−0.1978	−0.2047	−0.1494	−0.1492	−0.1880	−0.1947
17	−0.2006	−0.2000	−0.1811	−0.1821	−0.2092	−0.2123
18	−0.1764	−0.1861	−0.1543	−0.1585	−0.1927	−0.1885
$e_{j}$	0.9688	0.9749	0.9916	0.9932	0.9900	0.9876
$d_{j}=\left|1-e_{j}\right|$	0.0312	0.0251	0.0084	0.0068	0.0100	0.0124
$w_{j}=\frac{d_{j}}{\sum_{j=1}^{\ddot{n}}d_{j}}$	0.3323	0.2670	0.0892	0.0725	0.1071	0.1319
Weight values %	33.23	26.70	8.92	7.25	10.71	13.19

**Table 6:** Performance scores, S/N ratios, and rankings of the alternatives.

Trial No	$k_{ia}$	$k_{ib}$	$k_{ic}$	$k_{i}$	Ranking	$k_{i}$-S/N
1	0.0603	5.9837	0.8659	2.982	8	9.490
2	0.0662	7.8149	0.9517	3.734	4	11.443
3	0.0696	9.0049	1.0000	4.214	1	12.494
4	0.0496	3.4242	0.7121	1.890	15	5.529
5	0.0590	5.5103	0.8473	2.789	9	8.909
6	0.0639	6.9937	0.9175	3.401	5	10.632
7	0.0373	2.2020	0.5355	1.278	17	2.131
8	0.0551	4.4662	0.7919	2.351	11	7.425
9	0.0322	2.5186	0.4627	1.339	16	2.536
10	0.0672	8.1630	0.9661	3.875	3	11.765
11	0.0630	6.8073	0.9047	3.321	6	10.425
12	0.0675	8.2573	0.9696	3.913	2	11.850
13	0.0549	4.3950	0.7890	2.322	12	7.317
14	0.0616	6.2692	0.8847	3.104	7	9.838
15	0.0571	4.9710	0.8199	2.564	10	8.178
16	0.0504	3.4921	0.7243	1.926	14	5.693
17	0.0313	2.0659	0.4502	1.157	18	1.267
18	0.0540	4.2391	0.7759	2.252	13	7.051

**Table 7:** ANOVA results for *k_i_*.

Factors	DF	Seq SS	Adj MS	F-Value	*p*-Value	PCR %
Vc (m/min)	1	0.0116	0.01155	0.54	0.478	0.08
f (mm/rev)	2	11.4789	5.73947	269.87	*p* < 0.001	74.85
PA (°)	2	0.9944	0.49720	23.38	*p* < 0.001	6.49
RR (%)	2	2.6358	1.31792	61.97	*p* < 0.001	17.19
Error	10	0.2127	0.02127			1.39
Total	17	15.3334				100
R^2^: 98.61%, R^2^ (adj): 97.64%, R^2^ (pred): 95.51%						

**Table 9:** Verification experiment results for *k_i_*.

Taguchi-Based Entropy–CoCoSo Optimization	Initial Parameter	Optimal Control Factors and Levels	
		Predicted	Experimental
Experimental condition	${Vc}_{1}f_{1}{PA}_{1}{RR}_{1}$	${Vc}_{2}f_{1}{PA}_{3}{RR}_{3}$	${Vc}_{2}f_{1}{PA}_{3}{RR}_{3}$
Fz (N)	400		104
Mz (Ncm)	67		34
Ra (µm)	5.463		2.674
Rz (µm)	29.188		15.446
DD (mm)	0.072		0.054
CD (mm)	0.030		0.017
$k_{i}$	2.982	4.41	4.206
Improvement in $k_{i}$ = 1.224			
Percentage improvement in $k_{i}$ = 41.05%			

**Table 10:** Symbols, descriptions, and values of the parameters used in the calculation of the confidence interval.

Order	Symbol	Description	Values
1	$F_{\ddot{\alpha }:1,{\;Sd}_{e}}$	F ratio 95% (from F table)	4.9646
2	$\ddot{\alpha }$	Significant level	0.05
3	${Sd}_{e}$	Degrees of freedom of error	10
4	${\bar{V}}_{e}$	Error variance	0.02127
5	$\bar{r}$	Number of replications for confirmation experiment	3
6	$n_{eff}$	Effective number of replications	2.25
7	n	Total number of experiments	18
8	$T_{dof}$	Total main factor degrees of freedom	7

## Data Availability

The original contributions presented in this study are included in the article. Further inquiries can be directed to the corresponding author.
